# Latent variables and structural equation models for longitudinal relationships: an illustration in nutritional epidemiology

**DOI:** 10.1186/1471-2288-10-37

**Published:** 2010-04-30

**Authors:** Michel Chavance, Sylvie Escolano, Monique Romon, Arnaud Basdevant, Blandine de Lauzon-Guillain, Marie Aline Charles

**Affiliations:** 1 Biostatistics, CESP Centre for research in Epidemiology and Population Health, U1018, Inserm; F94807, Villejuif, France; 2 Université Paris Sud 11, UMRS 1018, F-94807, Villejuif, France; 3 Nutrition Department, University Hospital, Lille F-59000, France; 4 INSERM, Nutrinomique U 872, Paris, France; 5 Université Pierre et Marie Curie - Paris 6, Faculté de Médecine, Paris, France; 6 Endocrinology and Nutrition Department, Pitié-Salpêtrière Hospital, Paris, France; 7 Epidemiology of diabetes, obesity and chronic kidney disease over the life course, CESP Centre for research in Epidemiology and Population Health, U1018, Inserm, F-94807, Villejuif, France

## Abstract

**Background:**

The use of structural equation modeling and latent variables remains uncommon in epidemiology despite its potential usefulness. The latter was illustrated by studying cross-sectional and longitudinal relationships between eating behavior and adiposity, using four different indicators of fat mass.

**Methods:**

Using data from a longitudinal community-based study, we fitted structural equation models including two latent variables (respectively baseline adiposity and adiposity change after 2 years of follow-up), each being defined, by the four following anthropometric measurement (respectively by their changes): body mass index, waist circumference, skinfold thickness and percent body fat. Latent adiposity variables were hypothesized to depend on a cognitive restraint score, calculated from answers to an eating-behavior questionnaire (TFEQ-18), either cross-sectionally or longitudinally.

**Results:**

We found that high baseline adiposity was associated with a 2-year increase of the cognitive restraint score and no convincing relationship between baseline cognitive restraint and 2-year adiposity change could be established.

**Conclusions:**

The latent variable modeling approach enabled presentation of synthetic results rather than separate regression models and detailed analysis of the causal effects of interest. In the general population, restrained eating appears to be an adaptive response of subjects prone to gaining weight more than as a risk factor for fat-mass increase.

## Background

Structural equation and latent variable models [1,2] have previously been used in several fields of epidemiology. However, because the introduction of a latent variable becomes relevant as soon as a risk factor of interest cannot be obtained with a single exact measurement, it should be more popular. Structural equations allow modelling of different types of correlations between observations, regardless of their source (e.g., causal relationship, multiple outcomes, repeated measurements, longitudinal designs, etc). This approach is useful for path analysis, which, for example, enables separation of direct and indirect effects, and expands causal interpretations through the identification or elimination of potential mediators. Except for a few fields, like quality of life, psychometrics, socio-economics or dietary-intake assessments, in which the common problem is how to deal with psychometric properties of the questionnaires, these techniques remain seldom used by epidemiologists [3-7]. The aim of this paper is to encourage use of this approach. As an illustration, we applied it to data from a longitudinal study, previously analyzed with conventional regression models, about restrained eating as a risk factor for weight gain over a 2-year period, in a sample of adults from the general population [8]. Restrained eating [9], which has been described as the tendency to consciously restrict food intake to control body weight or promote weight loss, might have the paradoxical effect of inducing increased adiposity, through frequent episodes of loss of control and disinhibited eating. In this analysis, different indicators of adiposity were considered because no perfect measurement of adiposity is applicable for large epidemiological studies. Adiposity is often estimated through body mass index (BMI), but it can also be appreciated through determination of other fat-mass indicators, such as waist circumference, skinfold thickness and percent body fat, estimated with a bioimpedance analyzer. None of them provides an error-free assessment of global adiposity, but each one provides some information about body fat mass. If one tests separately the effect of restrained eating on each measurement, the familywise error rate [10], i.e. the probability of making any error in this family of tests when restrained eating has no effect on adiposity, is higher than the size of each test. By contrast, combining the four measurements into an adiposity latent variable within a structural model avoids the drawbacks of either arbitrarily choosing a single adiposity measurement or performing separate analyses on each fat-mass indicator. The results obtained with this novel analytical approach, using structural equation models and considering latent variables to model global adiposity, have been compared to those obtained with separate linear regressions.

## Methods

### Data

The dataset is a sample of the community-based Fleurbaix Laventie Ville Santé Study II (FLVS II), whose general aim was to investigate, in the general population, risk factors for weight and adiposity changes. The results of several cross-sectional studies suggested a link between restrained eating and weight gain, but those findings remain controversial. An aim of FLVS II was to measure longitudinally the effect of restrained eating on fat-mass changes and the effect of fat-mass on restrained eating changes.

Details concerning FLSV II study design and data collection can be found elsewhere [8]. Briefly, a first study, FLVS I [11] had been conducted on the children of all 579 families who had at least one child in primary school in 1992 in Fleurbaix or Laventie. Participation in FLSV II was proposed to 393 families who had not moved and who could be contacted in 1999: 294 families were recruited on a voluntary basis. Parents' overweight status and the subjects' ages and sexes, did not differ significantly between families who accepted to participate or not.

In our analysis, anthropometric data (weight, height, waist circumference, the bicipital, tricipital, subscapular and suprailiac skinfold thicknesses and percent body fat determined using a Tanita TBF 310 tetrapolar foot-to-foot bioimpedance analyzer) were collected by trained technicians at baseline and 2 years later, i.e., in 1999 and 2001. We used the sum of the four skinfold thicknesses as an indicator of the subcutaneous fat mass, named "skinfold thickness" for short in the following. Eating behavior was assessed using a French translation of the Three Factor Eating Questionnaire Revised 18-item version (TFEQ-R18) [12]. We focused on the cognitive restraint scale (CRS) of the eating-behavior questionnaire for the parents. The analyzed sample was composed of 256 females and 201 males.

### Latent variables and structural equation modeling

We briefly recall here the principle of this approach. Latent variables are used to translate the fact that several observed variables (also named manifest variables) are imperfect measurements of a single underlying concept. Each manifest variable is assumed to depend on the latent variable through a linear equation. The coefficients linking the latent and manifest variables are called loadings. A measurement scale has to be chosen for the latent variable. By convention, it is generally the scale of the first manifest variable, implying that the first loading is not estimated but fixed at 1. Because the indicators of the manifest variables are measured on various scales, it is useful to consider standardized estimates rather than raw loadings, using the observed standard deviations as measurement units for latent and manifest variables.

In structural equation modeling, relationships may be assumed between all manifest and latent variables according to acquired knowledge. These relationships are also defined through linear equations and a given variable can appear explanatory in one or several equations and as the outcome in another. As a result, it is possible to distinguish direct and indirect effects between an explanatory variable X and an outcome Y. When X has a causal effect on M, which causally influences Y, part or all of the effect of X on Y can be explained by the path X → M → Y, and M is called a mediator. The indirect effect of X on Y through M is obtained as the product of the estimated coefficients associated with the two arrows in the path. The regression coefficients and the variances of the residual errors that appear in the linear equations of the structural model specify how the manifest variables vary together. When they can be identified, they are estimated by optimizing a measure of adequacy between the observed and the model-predicted variance-covariance matrix (e.g. maximizing a likelihood).

### Fitted model

To validate the use of a latent variable approach, we fitted preliminary latent variable models to the four baseline anthropometric measurements (BMI, waist circumference, sum of skinfolds, percent body fat) to create a measurement model, as only one latent variable and its four manifest variables assessments are considered. We fitted such a model separately to measurements at baseline and two years later, first for the two sex groups, then for the entire sample. We also considered measurement models for the baseline measurements and their two-year changes explained by the baseline adiposity and its two-year change and we assumed the same relationships between latent adiposity and its four indicators at baseline and two years later; this model constrained the four loadings, i.e. the regression coefficients, to be identical for baseline adiposity, adiposity two years later and adiposity change (see appendix I). We considered variation rather than final values to avoid the problems of estimation and interpretation of coefficients issued from highly correlated variables [13].

**Figure 1:**
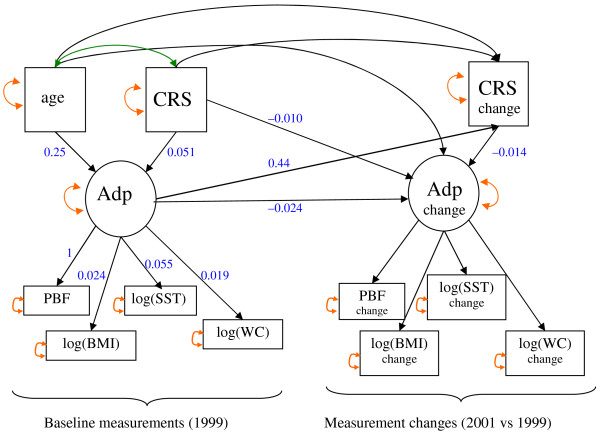
**Latent variable model for adiposity and restrained eating relationships**. Latent variables are represented by circles and manifest variables by rectangles. Single-headed arrows correspond to linear effects and double-headed arrows correspond to residual errors (orange lines) or covariance (green line). The values in blue are parameter estimates for the female group. Abbreviations: Adp (adiposity), CRS (cognitive restraint score), PBF (percent body fat), BMI (body mass index), ST (skinfold thickness), WC (waist circumference). For a detailed explanation of the model, see Appendix I.

Second, we fitted a structural equation model, adapted to the longitudinal design of our dataset and the specific epidemiological questions of interest. The diagram of this model is shown in Figure 1, where baseline adiposity is modeled marginally, while the effect of adiposity and CRS changes are adjusted for their baseline values (i.e., both the baseline value and its change appear in the same equation); CRS change was also assumed to depend on age, and adiposity change was assumed to depend on age and CRS change. Because the follow-up was constant (2 years), only age at entry was considered. Unmeasured confounders influencing both CRS and adiposity are not represented on this diagram, but are likely to be involved, biasing the cross-sectional association between baseline CRS and adiposity.

By contrast, the effect of baseline adiposity on CRS change was adjusted for baseline CRS and thus freed, at least partially, from the factors confounding the cross-sectional effect. Testing whether this effect is null can provide an answer to the question: *Does initial adiposity predict variation of CRS over time? *The direct effects of baseline CRS on adiposity and CRS changes were also adjusted for baseline adiposity and freed, at least partially, of the cross-sectional confounding effects. However, according to the orientation of the arrows, there are three paths from baseline CRS to adiposity change: the direct one and two indirect paths, one through CRS change and one through baseline adiposity. Thus, both the direct effect of baseline CRS on adiposity change and its indirect effects have to be considered to answer the second question: *Could restrained eating induce an increase of adiposity over time*? The indirect effect through baseline adiposity is not free of the confounding effects and does not have to be considered. The indirect effect through CRS change can be interpreted as a consequence of the change of intake. Note that since the measurement error on a baseline value also appears, with a minus sign for the corresponding change, the baseline value and its change will be negatively related, even in the absence of a causal link between the error-free baseline value and the error-free change. Appendix I provides a short formal presentation of the model.

All statistical analyses were performed on SAS9.1, using CALIS procedure. We log-transformed BMI, skinfold thickness and waist circumference to normalize their distributions and checked with Q-Q plots and Kolmogorov-Smirnov statistics that the transformed variables did not depart significantly from normal distributions. We chose to maximize the normal-theory maximum likelihood criteria. Among the various assessment of fit criteria, we focused on the root mean squared error of approximation (RMSEA) [14] and on the normed fit index (NFI) [15]. These criteria range from 0 to 1, with RMSEA close to 0 and NFI close to 1 for a correct fit. In order to build confidence intervals for indirect effects estimates or for the sum of direct and indirect effects, their variances were obtained by bootstrapping the sample subjects. A large number of bootstrap samples (1, 000) were used, to assess visually the assumed normal distribution of the estimators.

## Results

### General characteristics of the dataset

Characteristics of the sample are shown in Table 1. The four anthropometric measurements differed significantly according to sex, but not always in the same direction, namely percent body fat and skinfold thickness were higher for females than males, but BMI and waist circumference were higher for males. These differences suggest a different measurement model should be used for males and females. The CRS were clearly higher for females than males.

**Table 1:** Characteristic of the Studied Population

	Males n = 201	Females n = 256	p
Age in 1999 (yr)	44.0 (4.9)	42.4 (4.5)	<0.001
**1999 evaluation**			
Percent Body Fat (%)	23.0 (6.2)	33.2 (7.1)	<0.001
Body Mass Index (kg/m^2^)	25.7 (3.4)	24.7 (4.6)	<0.005
Skinfold Thickness (mm)	58.6 (25.2)	75.0 (32.2)	<0.001
Waist Circumference (cm)	91.6 (10.4)	79.4 (11.7)	<0.001
Cognitive Restraint Score	21.8 (18.2)	39.6 (21.4)	<0.001
**2001 evaluation**			
Percent Body Fat (%)	21.9 (6.1)	31.9 (7.6)	<0.001
Body Mass Index (kg/m^2^)	25.9 (3.5)	25.0 (4.8)	0.026
Skinfold Thickness (mm)	61.5 (25.3)	78.2 (34.2)	<0.001
Waist Circumference (cm)	91.5 (9.8)	79.6 (11.9)	<0.001
Cognitive Restraint Score	26.9 (19.7)	40.4 (21.3)	<0.001

Data are means (standard deviations). Differences according to sex were tested with Student's t-tests.

### Measurement model for adiposity and adiposity change

Results are given in Table 2. Analyses by sex showed that the covariations of the four baseline anthropometric measurements were correctly explained by latent adiposity with RMSEA between 0.00 and 0.16 and NFI between 0.97 and 0.99. The coefficients of determination R^2^, i.e., the squared standardized coefficients and the percentages of variance of each measurement explained by the latent variable were 0.65 for male skinfold thickness, both in 1999 and 2001. They were larger (between 0.83 and 0.96) in all other cases. The model did not fit as well the observations when all subjects were considered together, with RMSEA above 0.55 and NFI below 0.85, reflecting morphological differences between males and females, in addition to adiposity differences. Again this finding justifies the choice of running separate analyses for each sex. By contrast, the relationship between adiposity and anthropometric measurements can be expected to remain the same within each sex at baseline and 2 years later, and thus identical to the relationship between adiposity changes and measurement changes. Indeed, Table 2 shows that in each sex the loadings were similar in 1999 and 2001. This allowed us to impose equality constraints on these loadings and to consider models where the baseline measurements and their changes depended on the baseline adiposity. The model fits for the baseline measurements and their changes were only slightly modified when using constrained estimates in place of the specific ones: the largest decreases were found for the latent adiposity change, with NFI decreasing from 0.98 to 0.96 among males and from 0.99 to 0.96 among females. The loadings under equality constraints and the standardized coefficients are reported for each sex in Table 3.

**Table 2:** Measurement models for 1999 and 2001 evaluations: goodness of fit

	Males		Females	
	**1999**	**2001**	**1999**	**2001**

RMSEA*	0.00 [. ; 0.14]	0.05 [. ; 0.17]	0.16 [0.08 ; 0.26]	0.07 [. ; 0.17]
NFI**	0.999	0.997	0.988	0.996

R^2^*** Percent Body Fat	0.90	0.88	0.95	0.91
R^2 ^Body Mass Index	0.96	0.93	0.96	0.96
R^2 ^Skinfold Thickness	0.65	0.65	0.87	0.88
R^2 ^Waist Circumference	0.83	0.90	0.94	0.94

*RMSEA: Root Mean square Error of Approximation and its 95% confidence interval when available

**NFI: Normed Fit Index

*** R^2^; coefficient of determination

**Table 3:** Global measurement Model: Standardized Loadings of the Two Latent Variables

	Males				Females			
**Manifest variable**	**Estimate**	**Standard Error**	**Baseline Standardized Estimates**	**Change Standardized Estimates**	**Estimate**	**Standard Error**	**Baseline Standardized Estimates**	**Change Standardized Estimates**

Percent Body Fat	1	-	0.947	0.673	1	-	0.955	0.603
Body Mass Index	0.022	0.00064	0.976	0.942	0.024	0.00066	0.956	0.996
Skinfold Thickness	0.061	0.0033	0.815	0.357	0.055	0.0021	0.879	0.558
Waist Circumference	0.017	0.00068	0.912	0.546	0.019	0.00056	0.938	0.647

### Longitudinal modeling of adiposity and restrained eating

Concerning the global fit of the model, RMSEA and its 95% confidence interval was 0.11 [0.093 ; 0.014] for females and 0.16 [0.14 ; 0.18] for males, while their respective NFI were 0.91 and 0.84. The regression coefficients for the four baseline anthropometric measurements on baseline adiposity and of the four measurement changes on adiposity change, i.e., the loadings, are given in Table 3. The standardized coefficients showed that BMI was the most highly correlated and skinfold thickness was the least correlated to the latent variables. The standardized coefficients of percent body fat, skinfold thickness and waist circumference were clearly lower for changes than for baseline measurements (around 0.6 or lower versus 0.9). On the other hand, the four BMI standardized coefficients were quite high (between 0.94 and 1.00).

Regression coefficient estimates of the structural model are summarized in Table 4. For males as for females, both baseline CRS and age were positively related to baseline adiposity. CRS changes depended significantly on the baseline adiposity: 95% confidence interval (CI_95_) = [0.18 - 0.70] for females and [0.22 - 0.94] for males; subjects of either sex with high baseline adiposity were more likely to increase their CRS over time. As expected, adiposity and CRS changes were negatively related to the corresponding baseline value, although the relationship was not significant for female adiposity.

**Table 4:** Structural Equation Model: Regression Coefficients

		Males			Females		
**Outcome Variable**	**Explanatory Variables**	**Estimate**	**Standard Error**	**t value**	**Estimate**	**Standard Error**	**t value**

Baseline Adiposity	Baseline Age	0.195	0.082	**2.36**	0.254	0.096	**2.66**
	Baseline CRS	0.084	0.022	**3.72**	0.051	0.020	**2.56**

Adiposity Change	Baseline Adiposity	-0.044	0.022	**-2.03**	-0.024	0.021	-1.17
	Baseline Age	-0.018	0.025	-0.74	0.038	0.030	1.24
	Baseline CRS	0.012	0.007	1.67	-0.010	0.007	-1.39
	CRS Change	-0.011	0.008	-1.28	-0.014	0.010	-1.45

CRS Change	Baseline Adiposity	0.577	0.183	**3.16**	0.438	0.134	**3.28**
	Baseline Age	0.392	0.210	1.88	0.023	0.200	0.12
	Baseline CRS	-0.342	0.058	**-5.90**	-0.286	0.042	**-6.89**

The model assumed that one direct and two indirect effects of baseline CRS could explain adiposity changes. Table 5 gives their estimates. The distribution of the bootstrapped estimates looked normal and, for the direct effects, the asymptotic and bootstrap standard error estimates were consistent. The indirect effects of baseline CRS on adiposity change through CRS change were estimated as 0.004 (CI_95 _= [-0.002 - 0.009]) for males and 0.004 (CI_95 _= [-0.002 - 0.010] for females. The sum of this indirect effect and the direct one was estimated as 0.016 for males (CI_95 _= [0.003 - 0.029]) and -0.006 for females (CI_95 _= [-0.018 - 0.007]).

**Table 5:** Direct and Indirect Effects of Baseline CRS on Adiposity Change

effect	Males		Females	
	**Estimate**	Standard error*	**Estimate**	Standard error*

1 (direct)	0.012	0.0070	-0.0096	0.0069
2 (indirect through CRS change)	0.0036	0.0028	0.0040	0.0031
3 (indirect through baseline adiposity)	-0.0037	0.0021	-0.0012	0.0011
1+2 (partial)	0.016	0.0064	-0.0056	0.0064
1+2+3 (total)	0.012	0.0065	-0.0068	0.0064

*Obtained by bootstrapping the sampled subjects 1000 times

### Comparison with usual linear regressions

If we had not used a latent variable approach, we would have fitted several regression models to study the longitudinal effect of eating restriction on adiposity. In particular the CRS change would have been separately regressed on each baseline anthropometric measurement, adjusting for the same explanatory variables as in the structural model. For instance, one can estimate the coefficients of a linear regression explaining how the percent body-fat change depends on its baseline value, age and change in CRS. Table 6 reports the estimates of the coefficients linking the four changes of adiposity indicators to their baseline values and their counterpart in the latent variable model. Results were consistent, with all coefficients significantly positive and Wald test values (coefficient/standard error) around 3. Similarly, it would be possible to regress any change of a given manifest variable on baseline CRS, adjusting for its baseline value, age and CRS change. The obtained coefficient would be directly comparable to the corresponding direct effect obtained in our analysis, but that simple regression approach would not provide any indirect effect.

**Table 6:** Comparisons of Approaches with and without Latent Variables to Study the Effect of Baseline Fat Mass Measurements on CRS Change

	Fat mass measurement	Regression coefficient of CRS change on baseline measurements *	
		
		Males	Females
Manifest	Percent Body Fat	0.45 (0.17)	0.40 (0.13)
	Body Mass Index	26.3 (8.1)	18.2 (5.2)
	Skinfold Thickness	8.1 (2.5)	7.0 (2.1)
	Waist Circumference	25.1 (9.6)	16.3 (6.6)

Latent variable	Adiposity	0.58 (0.18)	0.44 (0.13)

* Regression coefficient estimates (standard error) adjusting for age and baseline CRS.

## Discussion

### Latent variables and measurement model

When fitting longitudinal models for adiposity and restrained eating, both goodness of fit criteria, RMSEA and NFI, worsened in comparison to the model fits of the measurement models obtained separately with the four baseline anthropometric measurements and the four measurement changes. That observation means that the relationships between each of the four indicators and its change cannot be reduced to the relationship between baseline adiposity and its change. Each of the four anthropometric indicators provides an imperfect assessment of global adiposity: BMI, because it also includes lean body mass, and the other three because they reflect local components of total fat mass: mainly the lower part of the body for percent body fat by Tanita bioimpedancemetry, abdominal compartment for waist circumference, and subcutaneous compartment for skinfold thicknesses. Adiposity changes may preferentially affect a given compartment for some subjects and another one for other other subjects. Similarly, the effect of the explanatory variables on the indicators cannot be reduced to their effect on latent adiposity. For example, age may affect BMI, through modifications of fat mass and lean mass. However, the used model provided a reasonable fit and was able to answer the epidemiological questions of interest.

### Comparison of statistical approaches

When studying a latent change, some authors prefer to use as manifest variables the baseline measurements and the time 2 measurements rather than the baseline measurements and their changes [16,17]. Under the equality constraint on the loadings at baseline and at time 2, both measurement models are similar (see appendix I). They differ, however, for the residual errors which should be equal or almost equal at time 1 and time 2 for any raw measurement but are different for a baseline measurement and its change. For each sex, we verified that in the measurement models, the loadings and the fit indices were similar when using either parameterization with and without the equality constraints.

What are the pros and cons of a latent variable analysis, as compared with separate analyses on each indicator? A latent variable analysis considers a combination of the four measurements which expresses what makes them vary together, global adiposity. Thus, it allows a synthetic presentation of results while improving precision, reducing the number of tests and limiting multiple testing difficulties. Here, each of the individual measurement analyses gave similar conclusions, which were the same as that obtained with the latent variable approach. Clearly, this cannot be always the case. When individual analyses are not consistent, a latent variable model provides an easily interpretable synthesis. Moreover, a by-product of our latent variable approach was that, among the four fat-mass indicators, BMI was the closest to latent adiposity for baseline measurement and, especially, for 2-year changes. When a single measurement exhibits a relationship with the latent variable as strong as BMI, there is not much to gain by considering other measurements; but should one decide to consider several measurements, we recommend a latent variable rather than separate analyses of each indicator.

Structural equation and path analyses are very useful for causal interpretation. Of course, the interpretations are conditional on the validity of the assumed model. Physiologically, the short-term effect of restrained eating is decreased adiposity. However, at baseline, high CRS were associated with high adiposity in each sex group. This cross-sectional association is insufficient to establish a long-term causal link between restrained eating and adiposity. The most likely explanation is that this association is confounded by some subjects' propensity to easily gain weight and their efforts to counterbalance this tendency through restrained eating. Accordingly, the longitudinal part of the model showed that, adjusting for baseline CRS, subjects with a high initial adiposity had a larger CRS increase during the 2-year follow-up than the others. The direct effect of baseline CRS on adiposity change was not significant for either sex, and of opposing signs for males and females. Practically, for a given sex, a CRS 20 units above the mean implied an expected BMI change of exp(20 × CRS effect on adiposity × loading of log(BMI)), respectively exp(-20 × 0.096 × 0.024) = 0.995, i.e., a decrease of 0.5% for females, and exp(20 × 0.012 × 0.022) = 1.005, i.e., an increase of 0.5% in males. The indirect effect of baseline CRS through CRS change was positive but small for each sex (0.004). The indirect effect through baseline adiposity is difficult to interpret because it relies on the strongly confounded cross-sectional association. In any case, its estimates were negative for females (-0.001) and males (-0.004). Finally, the longitudinal effect of baseline CRS, free of the cross-sectional confounding factors, is the sum of the direct effect and of the indirect effect through CRS change. The estimate for males was significantly positive (+0.016) but non significant of opposite sign (-0.006) for females. The effect observed for males was found significantly positive, however we considered that the direct effect of CRS on adiposity change (adjusted for CRS change) provide the best measurement of the effect of CRS on adiposity change. The indirect effect through CRS change is at least partly due to the regression to the mean (the expected negative relationships between baseline CRS and CRS change) and to the physiologic effect of CRS change on adiposity change. The relationships observed between each baseline value and its change were negative, as expected, although only three of them were significant, probably because of limited statistical power.

Cross-sectional studies have shown that restrained eating is frequent in those with high adiposity [18-20]. The results of prospective studies are more controversial. Higher restraint scores were associated with better weight maintenance after weight loss [21] or weight gain [22] prevention intervention. In the general population, Drapeau et al [23] found that initial restrained eating was related to subsequent weight gain positively in women but negatively in men, which is the opposite of our results. Hays et al [24] found that restraint was protective against weight gain only in women with high levels of disinhibition. That latter study was retrospective and self-reporting of past body weight may have biased past relationships. In adults with a familial history of obesity, non-obese women with the highest CRS were those who had been obese in childhood or adolescence, suggesting a beneficial effect of cognitive restriction for weight control in these women [25]. Altogether, we do not consider that available data from general population supports the hypothesis that restraint eating could induce an increase in adiposity: i) because of the inconsistency between studies ii) because of the inconsistency of the relationships observed according to sex; iii) because of the low level of significance of the observed relationship (p = 0.05 for males in our study).

## Conclusions

This latent variable and structural equation model enabled us to present synthetic results rather than four separate analyses for each sex group and to perform a detailed analysis of the causal mechanisms involved. It confirmed our previous observations; in the general population, restrained eating appears to be more of an adaptive response of subjects prone to gaining weight than a risk factor for increased fat mass.

## Competing interests

The authors declare that they have no competing interests.

## Authors' contributions

SE and MC developed the model, performed all statistical analyses and participated to article writing. BdLG and MAC were involved in all study aspects from its conception to article writing. MR and AB participated in the conception and design of the FLVS study and reviewed the article. All authors approved the final manuscript.

## Appendix I: Latent Variables and Structural Equation Model

Each arrow in the diagramed model (Figure 1) has an equation counterpart. Let *A*^*k *^denotes the latent variable baseline adiposity (*k *= 0) or adiposity change (*k *= 1),  denotes the *i*^th ^indicator of the latent variable *A*^*k *^(*i *= 1...4, for the four anthropometric measurements), i.e., the *i*^th ^baseline measurement (*k *= 0) or the 2-year change in the *i*^th ^measurement (*k *= 1), and *Z*_*j *_denotes the *j*^th ^explanatory variable, age (*j *= 1), baseline CRS (*j *= 2) or CRS change (*j *= 3). The measurement model specifies the relationships between the two latent variables and their four indicators, displayed on the lower part of the diagram; it is expressed with the following equations:

where the residual errors,  (for *i *= 1...4, and *k *= 0, 1) are Gaussian random variables with null expectation. The saturation *λ*_*i *_is the regression coefficient of the *i*^th ^manifest variable for the corresponding latent variable. Note that, in agreement with the assumptions used in our analysis, the same four loadings are used for both latent variables. A consequence of this constraint is that the model can be reparameterized as

This is the model and the parameterization used in the article. An alternative model uses two different sets  for the baseline adiposity (*k *= 0) and the adiposity change (*k *= 1). The coefficient *λ*_1_, linking the first manifest variable (here, percent body fat) to its latent variable, is not estimated but fixed at 1. As a result, latent adiposity is arbitrarily expressed on the same measurement scale as percent body fat. Because the latent variable indicators are measured on various scales, it is useful to consider standardized estimates rather than raw loadings, using the observed standard deviations as measurement units for latent and manifest variables, namely .

Note that, for a given *λ*_*i *_obtained under equality constraints, there are two standardized coefficients, one for each latent variable.

The structural model specifies all the relationships between the explanatory variables and the outcomes of interest, displayed on the upper part of the diagram; it is expressed with

where the residual errors, *ζ*^*k*^(*k *= 0, 1) and *ζ*_3 _are Gaussian random variables with null expectation. To simplify the equations, we centered all observed variables, so that intercepts no longer appear.

## Pre-publication history

The pre-publication history for this paper can be accessed here:

http://www.biomedcentral.com/1471-2288/10/37/prepub
